# Experimental evaluation of a GPU‐based Monte Carlo dose calculation algorithm in the Monaco treatment planning system

**DOI:** 10.1120/jacmp.v17i6.6455

**Published:** 2016-11-08

**Authors:** Moti R. Paudel, Anthony Kim, Arman Sarfehnia, Sayed B. Ahmad, David J. Beachey, Arjun Sahgal, Brian M. Keller

**Affiliations:** ^1^ Department of Medical Physics Sunnybrook Health Sciences Center Toronto ON Canada; ^2^ Department of Radiation Oncology University of Toronto Toronto ON Canada; ^3^ Department of Radiation Oncology Sunnybrook Health Sciences Center Toronto ON Canada

**Keywords:** Monte Carlo algorithm, GPUMCD, XVMC, Monaco TPS, radiotherapy

## Abstract

A new GPU‐based Monte Carlo dose calculation algorithm (GPUMCD), developed by the vendor Elekta for the Monaco treatment planning system (TPS), is capable of modeling dose for both a standard linear accelerator and an Elekta MRI linear accelerator. We have experimentally evaluated this algorithm for a standard Elekta Agility linear accelerator. A beam model was developed in the Monaco TPS (research version 5.09.06) using the commissioned beam data for a 6 MV Agility linac. A heterogeneous phantom representing several scenarios — tumor‐in‐lung, lung, and bone‐in‐tissue — was designed and built. Dose calculations in Monaco were done using both the current clinical Monte Carlo algorithm, XVMC, and the new GPUMCD algorithm. Dose calculations in a Pinnacle TPS were also produced using the collapsed cone convolution (CCC) algorithm with heterogeneity correction. Calculations were compared with the measured doses using an ionization chamber (A1SL) and Gafchromic EBT3 films for $2\times 2\;{\text{cm}}^{2},5\times 5\;{\text{cm}}^{2}$, and $10\times 2\;{\text{cm}}^{2}$ field sizes. The percentage depth doses (PDDs) calculated by XVMC and GPUMCD in a homogeneous solid water phantom were within 2%/2 mm of film measurements and within 1% of ion chamber measurements. For the tumor‐in‐lung phantom, the calculated doses were within 2.5%/2.5 mm of film measurements for GPUMCD. For the lung phantom, doses calculated by all of the algorithms were within 3%/3 mm of film measurements, except for the $2\times 2\;{\text{cm}}^{2}$ field size where the CCC algorithm underestimated the depth dose by ∼5% in a larger extent of the lung region. For the bone phantom, all of the algorithms were equivalent and calculated dose to within 2%/2 mm of film measurements, except at the interfaces. Both GPUMCD and XVMC showed interface effects, which were more pronounced for GPUMCD and were comparable to film measurements, whereas the CCC algorithm showed these effects poorly.

PACS number(s): 87.53.Bn, 87.55.dh, 87.55.km

## I. INTRODUCTION

Monte Carlo (MC)‐based dosimetric calculations are considered the most accurate methods and are taken as gold standards in radiotherapy. Many MC packages such as EGSnrc,^(1)^ PENELOPE,^(2)^ MCNP,^(3)^ and GEANT4^(4)^ have been used and benchmarked with experimental data very accurately in a variety of clinical radiotherapy scenarios. There are a variety of MC‐based dose calculation algorithms used in radiotherapy treatment planning systems (TPS).^5^, ^6^, ^7^, ^8^ Generally, these algorithms make some sacrifices to gain computational efficiency required to achieve appropriate statistical uncertainty levels. To test the robustness of a MC‐based TPS, AAPM TG‐105 recommends^(9)^ commissioning the TPS using a range of field sizes (small to large), homogeneous water or water‐like phantoms, as well as heterogeneous phantoms containing lung‐ or bone‐like media.

Recently, a new commercial GPU‐based Monte Carlo dose calculation algorithm (GPUMCD)^10^, ^11^ has been proposed by the vendor Elekta (Elekta AB, Stockholm, Sweden) and is available in the research version of the Monaco TPS. The Monaco TPS currently has a clinical MC dose calculation algorithm called XVMC^(12)^ for photons, which is founded on the voxel‐based MC algorithm (VMC)^(13)^ for electron beams. The new GPUMCD algorithm is capable of modeling dose for both a standard linear accelerator and for an Elekta MRI linear accelerator where it can model magnetic field effects. In its current implementation within Monaco, GPUMCD specifies the materials for a voxel in a patient image to one of four materials (air, lung, soft tissue, and bone) based upon the physical density of the voxel. The relevant radiological properties and the cross‐sectional data for these materials are then taken from ICRU Report No. 46.^(14)^ These data are scaled and mapped for a voxel inside a patient based upon its physical density during the MC calculation. XVMC does not specifically assign a material type for a given voxel but uses the data from ICRU Report No. 46^(14)^ for a range of human anatomies to create a set of transfer functions that map the radiological data of water to the corresponding data for a material in a given voxel, based upon its physical density. Since GPUMCD uses specific material assignments, it may be more accurate than XVMC in the cases of high‐Z anatomies like bone if there are no material binning errors. However, this needs to be evaluated.

In the past, the experimental validation study of Monaco was limited to the XVMC algorithm.^15^, ^16^ Although there exists an evaluation study of a standalone version of the GPUMCD algorithm for IMRT optimization,^(17)^ no work has been done, to our knowledge, on the experimental evaluation of the GPUMCD algorithm implemented in the Monaco TPS.

This work is novel because we designed and fabricated a realistic heterogeneous phantom, simulated it using a clinical CT scanner with a clinical protocol, and evaluated this new GPUMCD algorithm in the Monaco TPS with measurements using film and ion chamber dosimetry for a range of field sizes. We also made the dosimetric comparison of GPUMCD among two existing clinical dose calculation algorithms (XVMC in Monaco and CCC in Pinnacle).

## II. MATERIALS AND METHODS

### A. Beam modeling in Monaco

A beam model was developed by the vendor Elekta in the Monaco TPS (v.5.09.06) using the commissioned beam data of a clinical 6 MV Agility linac in our center. The beam model uses a virtual source model for simulating the linac head. The beam model parameters are the same for both the XVMC and GPUMCD codes (the two Monaco MC codes differ only in the radiation transport calculations within the patient). These are the parameters for modeling the distribution of primary and scattered photons, as well as secondary charged particles in the MC simulation of the linac head. It also includes nominal beam energy and off‐axis profiles. There are quite a large number of parameters in the list provided by the vendor and may not be relevant for the paper. Tighter criteria (2% or 2 mm in high gradient [30%/cm] region, 2% elsewhere in the percent depth dose [PDD] and beam profiles, and 2% in output factors for homogeneous water medium) than the requirements of the Van Dyk criteria^(18)^ for matching the calculated data with the measured commissioned data was used by the vendor. The phantom size, dose calculation grid resolution, and the statistical uncertainty used by the vendor were in accordance with the recommendations of AAPM TG‐105.^(9)^

### B. Phantoms developed for measurements

**Figure 1:**
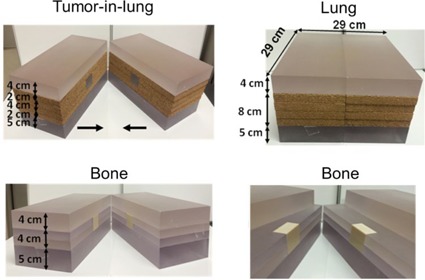
Various phantom configurations used in the study. Horizontal arrows in the tumor‐in‐lung phantom (left image in top row) indicate how we move two parts of the phantom and can place a film in between them at the center of the phantom. The central bone inserts are visible in the bone phantom when displacing the top layer of the phantom (right image in bottom row).

A homogeneous phantom was constructed by using Solid Water slabs from Gammex (Gammex Inc., Middleton, WI) of $30\times 2\;{\text{cm}}^{2}$ cross section and various thicknesses. Although the beam model was developed by using the commissioned water‐tank data, we initially compared the calculations in a homogeneous phantom against measurements to provide the validation of the film calibration and also to understand the inherent uncertainty level in the measurements. A complex heterogeneous phantom representing several scenarios — tumor‐in‐lung, lung alone, and bone‐in‐tissue — was designed and built using two sets of slabs of various thicknesses and materials (Fig. 1). The materials used included pure transparent polystyrene (ρ=1.04 g/cc) representing tissue or tumor, cork (ρ=0.19 g/cc) for lung, and cortical bone (ρ=1.79 g/cc) from Gammex RMI for bone. Polystyrene (PS) had similar attenuation properties to the solid water (this was verified with transmission measurements using an ion chamber, for the range of thicknesses used in the phantom, and they were within 0.2% of each other for a variety of field sizes and depths).

The heterogeneous phantoms were constructed with these materials in the following manner.

For the tumor‐in‐lung phantom, a 5 cm thick PS slab was placed as the bottom part of the phantom. Placed on top of this slab were four layers of cork, each 2 cm thick, with a centrally embedded $4\times 4\times 4\;{\text{cm}}^{3}$ PS “tumor” inside the cork. The PS tumor was sectioned into four $4\times 2\times 2\;{\text{cm}}^{3}$ pieces such that film may be inserted in the three orthogonal directions. The top of the phantom had a 4 cm thick PS slab.

As with the tumor‐in‐lung phantom, the lung phantom had the same bottom PS slab, central four layers of cork, and a top PS slab, with the difference being that the central part of the cork did not have a PS tumor embedded in it.

For the bone phantom, a 5 cm thick PS slab served as the bottom of the phantom, with four 2 cm thick PS layers on top of this bottom slab. The four PS layers had a central receptacle where a $4\times 4\times 4\;{\text{cm}}^{3}$ cortical bone was placed. As with the tumor‐in‐lung phantom, the bone was sectioned in four $4\times 2\times 2\;{\text{cm}}^{3}$ pieces so that film could be inserted. The top of the phantom was a 4 cm thick PS slab. Figure 1 shows the different heterogeneous phantoms used in the study.

### C. Measurements

#### C.1 Film

Film measurements were done using Gafchromic EBT3 films (Ashland, Covington, KY). A calibration curve was created for a given batch of film using the red channel with 16 points in the dose range of 0–10 Gy. Films were scanned on an Epson Expression 10000XL flatbed color image scanner (Seiko Epson Corp., Nagano, Japan) with 48‐bit color depth and 150‐dpi resolution. EBT3 film sheets $\left(12\times 10\;{\text{cm}}^{2}\right)$ were inserted in a Solid Water phantom $\left(30\times 30\times 32\;{\text{cm}}^{3}\right)$ at 1.5 cm depth and irradiated with an $8\times 2\;{\text{cm}}^{2}$ beam. An ion chamber measurement was also taken at 20 cm depth to account for the output variation of the linac for each film irradiation. To reduce the uncertainty in the calibration curve, three independent measurements were taken for each of the 0 Gy, 2 Gy, and 10 Gy dose levels. The vendor‐suggested method^(19)^ of using the rational function [$D(x)=(p_{1}\;x+p_{2})/(x+q_{1})$, where D is dose at a point in the film with pixel value x, and $p_{1},p_{2}$, and $q_{1}$are the fitted parameters] was followed for generating the calibration curve.

During the film measurements in a phantom, for each field size, three separate film irradiations were done and then averaged to reduce the statistical uncertainty. To further reduce the noise, the dose was averaged using a 1 mm grid resolution in the depth direction and 1.5 mm in the perpendicular direction. Finally, the normalized central axis (CAX) depth dose was calculated. Initially, we found a large (up to 6%) systematic deviation in the measured PDDs due to the concavity of the film. This problem was almost entirely removed by clamping the film edges during scanning using two polystyrene rulers. The overall uncertainty of the film measurements in this study was estimated to be <3% for a heterogeneous phantom and <2% for a homogeneous phantom. Three field sizes ($2\times 2\;{\text{cm}}^{2},5\times 5\;{\text{cm}}^{2}$, and $10\times 10\;{\text{cm}}^{2}$) with a 90 cm source‐to‐surface distance (SSD) were used for each phantom and 200 MUs were used for each irradiation. The orientation of the film was in the direction of the beam central axis, except in the bone phantom where the film was positioned at various depths perpendicular to the beam central axis. In the case of the bone phantom, we found that film measurements with orientation along the beam central axis resulted in a differential attenuation of the beam, but this situation was not possible to simulate properly in the TPS due to the size of the film thickness. The film thickness (∼272 micron) was smaller than the resolution of a CT image, as well as the smallest possible dose calculation grid resolution in the TPS.

#### C.2 Ionization chamber

Ion chamber measurements were made using an Exradin A1SL ionization chamber (Standard Imaging Inc., Middleton, WI). This ion chamber was chosen due to its small volume (0.053 cc) and small size (internal diameter of 4 mm), better suited for small field dosimetry. We used seven nominal depths (1.5, 5, 10, 13.5, 15, 20, and 25 cm) for the homogeneous Solid Water phantom and five depths (two depths at 1.5 and 2.5 cm before the start of a heterogeneous medium and three depths at 1.5, 2.5 and 3.5 cm downstream from the end of the heterogeneous medium) for the heterogeneous phantoms. Irradiation conditions were the same as in the film measurements. The depth ionization values were then shifted upstream by 1.2 mm to account for the effective point of measurement and normalized at nominal depth of dose maximum to get the PDD.

### D. Calculations in the TPS

Once the beam model was implemented, the MC transport was carried out within the patient image for calculating dose in Monaco. The Monaco TPS requires a “CT number vs. electron density relative to water” calibration table, whereas Pinnacle TPS uses a “CT number vs. physical density relative to water” calibration table. An RMI tissue characterization phantom from Gammex was scanned in a Phillips Brilliance 16‐slice Big Bore CT scanner (Philips Healthcare System, Cleveland, OH) to generate these tables. The phantoms (homogeneous and heterogeneous) were scanned on the same CT simulator in helical mode with the following settings: 120 kVp, 400 mAs/slice, $16\times 0.75\;{\text{mm}}^{2}$ collimation, 1.0 mm slice thickness, 0.942 pitch, and 1 s rotation time.

Dose calculations in Monaco were done using the smallest possible voxel size of $1\times 1\times 1\;{\text{mm}}^{3}$ with 0.5% statistical uncertainty. In the Pinnacle TPS (v.9.02), dose calculations were done using the collapsed cone convolution (CCC) algorithm with the lowest possible dose grid resolution $\left(1.5\times 1.5\times 1.5\;{\text{mm}}^{3}\right)$. The heterogeneity correction was applied during the calculations in Pinnacle. The calculated dose distributions, with a 1 mm dose grid resolution, were exported from both the TPSs and analyzed in MATLAB (MathWorks, Inc., Natick, MA). Dose calculations were made for $2\times 2\;{\text{cm}}^{2},5\times 5\;{\text{cm}}^{2}$, and $10\times 2\;{\text{cm}}^{2}$ field sizes at 90 cm SSD, identical to the measurement conditions. Finally, the PDDs were compared against the measurements (film and ion chamber) and the calculations (GPUMCD, XVMC, and CCC).

## III. RESULTS

The results are shown here by first discussing a budgeted uncertainty analysis for both the film and ionization chamber measurements, followed by the homogeneous and heterogeneous phantom results.

### A. Uncertainty analysis

**Figure 2:**
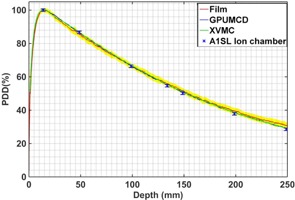
The percent depth‐dose curves (PDDs) in a homogeneous Solid Water phantom comparing the calculations by GPUMCD and XVMC with the film and ion chamber measurements for a $10\times 2\;{\text{cm}}^{2}$ field size.

**Figure 3:**
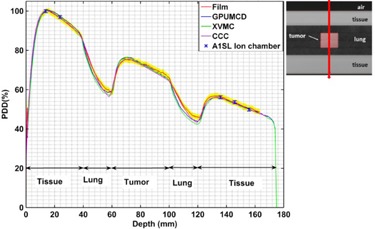
Variation of the PDDs in tumor‐in‐lung phantom comparing calculations with measurements for a $2\times 2\;{\text{cm}}^{2}$ field size. Inset shows a CT slice at CAX and the direction of the beam.

Tables 1 and 2 show the uncertainty budget analysis for film measurements and ion chamber measurements done for a homogeneous solid water phantom. All of the uncertainties noted in this work correspond to a 1‐sigma standard deviation (1 SD) around the mean. However, since the measurement reproducibility varies slightly with depth in the case of the PDD, the component of the overall uncertainty corresponding to measurement reproducibility in Tables 1 and 2 is calculated based on an average uncertainty of all discrete data points measured along the PDD curve. To avoid cluttering of the figures, we have presented the overall 1‐sigma uncertainty of the measurements (both for film and ion chamber) only in two representative cases: Fig. 2 for a homogeneous phantom and Fig. 3 for a heterogeneous phantom. For clarity, the uncertainty for film measurement is presented as a yellow envelope around the measured data, whereas error bars are used for the ion chamber measurement uncertainties.

**Table 1:** Uncertainty budget analysis for the PDD measurements using EBT3 film

*Description*		*Type A*	*Type B*
	$N_{D,W}$		0.7
	$k_{Q}$		0.6
Linac output (TG‐51)	Setup		0.6
	Other (chamber stability and correction factors)		0.4
	Chamber cross calib.		0.3
	Calib. curve fit		0.7
Measurement	Meas. reproducibility	1.169	
Positioning	Film alignment/setup		0.2
Overall uncertainty on PDD (1‐sigma)		1.83	

**Table 2:** Uncertainty budget analysis for the PDD measurements using the A1SL ionization chamber

*Description*		*Type A*	*Type B*
	Setup (ion chamber)		0.3
Measurement	Temp./press. variation Meas. reproducibility	0.3	0.34
	Normalization		0.54
Positioning	Phantom assembly		0.2
Overall uncertainty on PDD (1‐sigma)		0.77	

For film measurements, as noted in Table 1, the main components of the uncertainty budget are divided into four parts: linac output measurement (performed based on TG‐51 in this work), the film calibration curve (a.k.a. sensitometric curve) determination, the reproducibility of measurements, and the uncertainty introduced due to positioning and setup. The latter does take into account difficulties associated with sandwiching a large piece of film inside the phantom, as well as any other setup issues. Unlike Type A uncertainty, which is calculated through statistical analysis of measurements (such as standard deviation and standard error around mean of results), Type B uncertainty is calculated in this work through best scientific judgment and related literature.^20^, ^21^, ^22^

Table 2 shows the uncertainty budget for ionization chamber measurements. Some of the typical uncertainties that are often associated with chamber dose measurements are not included as they drop out due to the normalization of all points of a PDD curve by the maximum.

### B. Homogeneous phantom

Figure 2 shows the comparison of the PDDs for the homogeneous Solid Water phantom for a $10\times 2\;{\text{cm}}^{2}$ field size. Both the GPUMCD and XVMC results are within 1% of ion chamber measurements. Film measurement is within 2% or 2 mm of both the GPUMCD and XVMC calculations. Similar results were found for $2\times 2\;{\text{cm}}^{2}$ and $5\times 2\;{\text{cm}}^{2}$ field sizes but not shown here. “Within 2% or 2 mm” means (and hence for all such comparisons in this manuscript) that 100% of the points at depth >3 mm pass the well‐known gamma pass/fail criteria. The choice of 3 mm comes from the fact that the physical integrity of some of the films changed at the edges when cutting the original film into smaller piece and hence the dosimetric accuracy was compromised for up to the depth of 3 mm in the film measurements.

### C. Tumor‐in‐lung phantom

Figure 3 shows the comparison of the PDDs inside the tumor‐in‐lung phantom for a $2\times 2\;{\text{cm}}^{2}$ field size. The calculated dose is within 1% of ion chamber measurement and 2% or 2 mm of film measurements for all the algorithms outside the lung legion. The larger deviations are within the lung region. In the lung region, the calculated dose is within 2.5%/2.5 mm of film measurements for GPUMCD but both XVMC and CCC are different by up to 4% or 4 mm compared to the film measurements.

**Figure 4:**
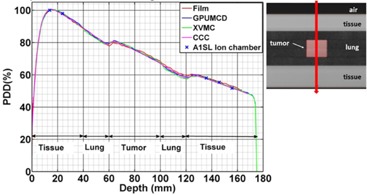
Variation of the PDDs in tumor‐in‐lung phantom comparing calculations with measurements for a $5\times 2\;{\text{cm}}^{2}$ field size.

**Figure 5:**
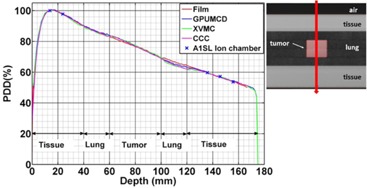
Variation of the PDDs in tumor‐in‐lung phantom comparing calculations with measurements for a $10\times 2\;{\text{cm}}^{2}$ field size.

Figures 4 and 5 show the comparison of the PDDs inside the tumor‐in‐lung phantom for $5\times 2\;{\text{cm}}^{2}$ and $10\times 2\;{\text{cm}}^{2}$ field sizes, respectively. The calculated dose is within 1% of ion chamber measurement and 2% or 2 mm of film measurement for all of the algorithms for both of these field sizes.

### D. Lung phantom

**Figure 6:**
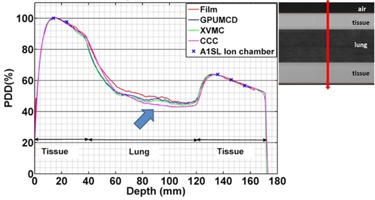
Variation of the PDDs in the lung phantom comparing calculations with measurements for a $2\times 2\;{\text{cm}}^{2}$ field size. A dose difference of ∼5% exists for CCC compared to the film measurements in the low‐dose gradient region inside lung indicated by an arrow.

Figure 6 shows the comparison of PDDs in the lung phantom for a $2\times 2\;{\text{cm}}^{2}$ field size. For all of the algorithms the calculated dose is within 1% of ion chamber measurement and 2% or 2 mm of film measurement, except in the lung region. In the lung region, the dose calculated by GPUMCD is within 3% or 3 mm of film measurements and the largest deviation is in the high‐dose‐gradient region immediately after entering the lung region from the tissue region. However, the difference gets progressively smaller in the low‐dose gradient region. On the other hand, CCC consistently underestimates the dose by about 5% in the low‐dose gradient region inside lung.

**Figure 7:**
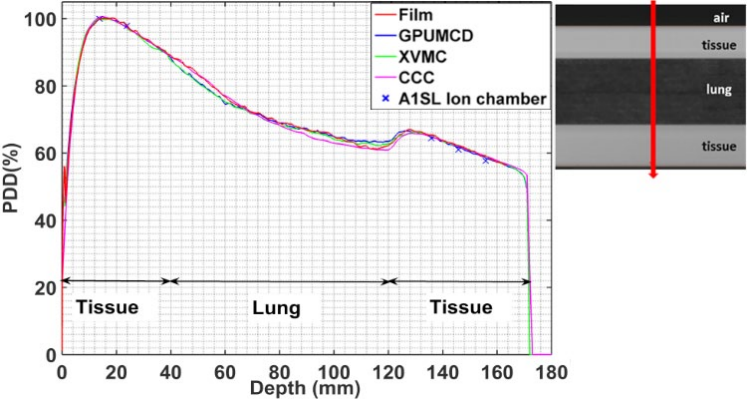
Variation of the PDDs in the lung phantom comparing calculations with measurements for a $5\times 2\;{\text{cm}}^{2}$ field size.

**Figure 8:**
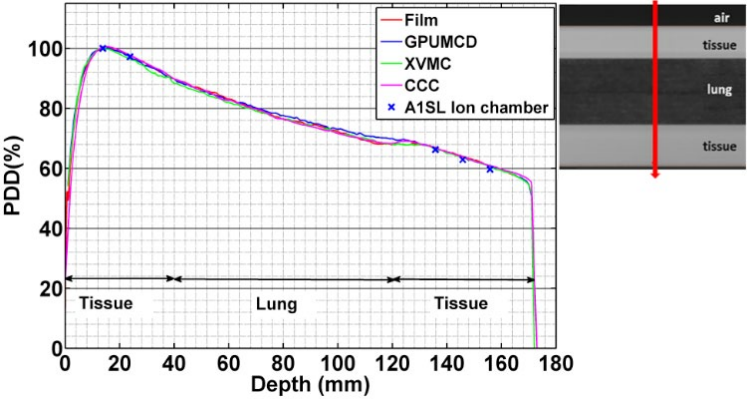
Variation of the PDDs in the lung phantom comparing calculations with measurements for a $10\times 2\;{\text{cm}}^{2}$ field size.

The calculated dose is within 1% of ion chamber measurement and 3% or 3 mm of film measurement in the lung phantom for all of the algorithms for the $5\times 2\;{\text{cm}}^{2}$ (Fig. 7) and $10\times 2\;{\text{cm}}^{2}$ (Fig. 8) field sizes.

### E. Bone phantom

**Figure 9:**
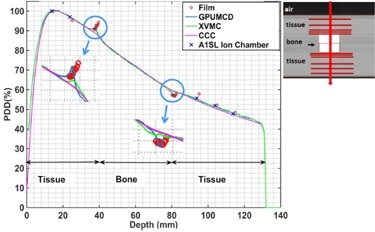
Variation of the PDDs in the bone phantom comparing calculations with film and ion chamber measurements for a $2\times 2\;{\text{cm}}^{2}$ field size. Inset shows a portion of a CT slice at CAX, the direction of the beam (vertical arrow) and schematic film positions (horizontal lines). The interface regions have been blown up for visualization.

Figure 9 shows the comparison of the PDDs through the bone phantom for a $2\times 2\;{\text{cm}}^{2}$ field size. The calculated dose for GPUMCD was within 1.0% of ion chamber measurement. Film measurements were within 2% or 2 mm of the GPUMCD calculations except at one point where film overestimated the dose by 2.6%. Film measurements show pronounced interface effects (the abrupt rise or fall of the PDD) on either side of the bone region. At the tissue–bone interface, the increase in dose, due to backscattering from bone, is pronounced for GPUMCD. The maximum dose at the interface in GPUMCD is ∼7% compared to the case without the bone (homogeneous phantom case) and occurs at about two voxels (2 mm) away from the true bone interface. The XVMC algorithm also shows this increase in dose, but the effect is not as pronounced as with GPUMCD. The CCC algorithm shows even less enhancement in dose at the interface. Interface effects at the bone–tissue interface (abrupt decrease in dose due to the lack of backscattering from the tissue side and then the rebuildup of dose) are also pronounced for GPUMCD, whereas for both XVMC and CCC this effect is almost negligible. Similar results were obtained for the $5\times 2\;{\text{cm}}^{2}$ and $10\times 2\;{\text{cm}}^{2}$ field sizes (data not shown).

## IV. DISCUSSION

In the more advanced radiation therapy techniques such as IMRT, VMAT, SBRT, or SRS, small field sizes and/or large doses per fraction are frequently used. Conventional dose calculation algorithms, including the CCC algorithm, are less accurate for these small field sizes as well as in a heterogeneous media like lung, bone or metals.^8^, ^23^, ^24^, ^25^ MC‐based dose calculation algorithms are considered to be the most accurate methods of dose calculation but there could be approximations used to make such algorithms faster and these can compromise the accuracy of the calculation. Hence, a thorough evaluation of MC algorithms is necessary for the various clinically relevant scenarios.

We evaluated the GPU‐based MC dose calculation algorithm (GPUMCD) in the Monaco TPS by comparing it against film and ion chamber measurements and also against two current clinical algorithms (XVMC and CCC) using our custom‐designed phantoms. These complex heterogeneous phantoms were designed to represent lung, tumor‐in‐lung, and bone‐in‐tissue. These are relevant phantom configurations to the heterogeneities found in a human body for the common radiation therapy sites. Our phantoms are versatile and allow film placement at different depths and orientations. Ion chamber measurements can also be made using the phantom. The dimensions of the central heterogeneous medium representing tumor or bone were chosen in such a way that the $2\times 2\;{\text{cm}}^{2},5\times 5\;{\text{cm}}^{2}$, and $10\times 2\;{\text{cm}}^{2}$ field sizes would be smaller than, just slightly bigger, and larger than the extent of the insert, respectively.

Comparison of the film and ion chamber measurements with the GPUMCD calculated dose in the homogeneous phantom provided the validation of the film calibration and also showed the inherent uncertainty level in the film measurements. Also, the GPUMCD algorithm calculated dose as accurately as the current clinical MC algorithm (XVMC).

In the heterogeneous tumor‐in‐lung or lung phantoms, the dose differences among all three algorithms were clinically insignificant (within 3% or 3 mm) and they were all within the uncertainty of the film and ion chamber measurements, except in the lung phantom where the CCC algorithm underestimated normalized depth dose by ∼5% compared to the film measurement for the smallest $\left(2\times 2\;{\text{cm}}^{2}\right)$ field size. In both of these phantoms, the largest deviation was in the lung region of the phantom. Although both the GPUMCD and XVMC algorithms were well within the uncertainty of the film measurements in these two phantoms, the systematic dose difference of up to 2% in the tumor‐in‐lung phantom and 3% in the lung phantom needs to be explored further. In a recent simulation study^(26)^ comparing the performance of a standalone GPUMCD code with GEANT4, similar underestimated results were found when the lung density of 0.26 g/cc was used in the GEANT4 simulations. The currently used radiological data in Monaco are obtained from ICRP‐46^(14)^ where the density of 1.05 g/cc is used for lung tissue. The radiological data for a lower‐density lung (the density of 0.19 g/cc used in our experiment) could be different from those used in Monaco. This difference, if present, could lead to a dosimetric difference. Further confirmation of this hypothesis would come after calculating dose in GEANT4 using a density of 1.05 g/cc and comparing the dose distribution with GPUMCD calculated in Monaco. We would like to leave this exploration study for future work.

For the heterogeneous bone phantom, the differences in the calculated dose among all the algorithms were clinically insignificant except at the tissue‐bone or bone‐tissue interfaces. Outside the bone region and its interfaces, both the ion chamber and film measurements agree with the calculations within their measurement uncertainties. The interface effects at the bone interfaces are more pronounced for GPUMCD, less pronounced for XVMC, and almost negligible for CCC. On either side of the bone, in the tissue region, GPUMCD results follow the film measurements well. The maximum dose at the tissue–bone interface (∼7%) is close to the film measurement and is also comparable to the published result^(27)^ of 8% for a polystyrene–bone interface. The pronounced interface effects in the case of GPUMCD come from its capability of modeling materials (including bone) individually, as stated in the introduction section. A further measurement study with a finer depth resolution inside the bone is needed to completely evaluate the accuracy of GPUMCD inside bone. This would require a major modification to the phantom. We also noticed that the maximum dose at the tissue–bone interface and the minimum dose at bone–tissue interface for GPUMCD occur at 1 or 2 voxels (1 or 2 mm) away from the actual interface. Due to the partial volume effect in the CT images, these tissue pixels have electron densities higher than that of tissue and are incorrectly mapped to bone material type during the dose calculation in Monaco. Last, although the GPUMCD algorithm has been developed for increased speed performance, this was not evaluated in this study.

## V. CONCLUSIONS

The new GPUMCD algorithm was evaluated against measurements and compared to the XVMC and CCC algorithms using heterogeneous phantoms for $2\times 2\;{\text{cm}}^{2}$ to $10\times 2\;{\text{cm}}^{2}$ field sizes. There were no clinically significant dose differences between the GPUMCD, XVMC, CCC algorithms and measurements for the lung or tumor‐in‐lung phantoms, except for the $2\times 2\;{\text{cm}}^{2}$ field size in the lung phantom where the CCC algorithm underestimated the PDD by ∼5% in the lung region compared to film measurements. In the bone phantom, all of the algorithms were equivalent except at the interfaces. Both the GPUMCD and XVMC algorithms showed the interface effects more pronounced and consistent for GPUMCD, whereas CCC showed the interface effects poorly.

## ACKNOWLEDGMENTS

We acknowledge the financial support of Elekta, AB, Stockhom.

## COPYRIGHT

This work is licensed under a Creative Commons Attribution 3.0 Unported License.
